# A review of statistical estimators for risk-adjusted length of stay: analysis of the Australian and new Zealand intensive care adult patient data-base, 2008–2009

**DOI:** 10.1186/1471-2288-12-68

**Published:** 2012-05-16

**Authors:** John L Moran, Patricia J Solomon

**Affiliations:** 1 Department of Intensive Care Medicine, The Queen Elizabeth Hospital, Woodville, SA 5011, Australia; 2 School of Mathematical Sciences, University of Adelaide, Adelaide, SA 5000, Australia; 3 Australian and New Zealand Intensive Care Society, Carlton Victoria, 3053, Australia

## Abstract

**Background:**

For the analysis of length-of-stay (LOS) data, which is characteristically right-skewed, a number of statistical estimators have been proposed as alternatives to the traditional ordinary least squares (OLS) regression with log dependent variable.

**Methods:**

Using a cohort of patients identified in the Australian and New Zealand Intensive Care Society Adult Patient Database, 2008–2009, 12 different methods were used for estimation of intensive care (ICU) length of stay. These encompassed risk-adjusted regression analysis of firstly: log LOS using OLS, linear mixed model [LMM], treatment effects, skew-normal and skew-t models; and secondly: unmodified (raw) LOS via OLS, generalised linear models [GLMs] with log-link and 4 different distributions [Poisson, gamma, negative binomial and inverse-Gaussian], extended estimating equations [EEE] and a finite mixture model including a gamma distribution. A fixed covariate list and ICU-site clustering with robust variance were utilised for model fitting with split-sample determination (80%) and validation (20%) data sets, and model simulation was undertaken to establish over-fitting (Copas test). Indices of model specification using Bayesian information criterion [BIC: lower values preferred] and residual analysis as well as predictive performance (R^2^, concordance correlation coefficient (CCC), mean absolute error [MAE]) were established for each estimator.

**Results:**

The data-set consisted of 111663 patients from 131 ICUs; with mean(SD) age 60.6(18.8) years, 43.0% were female, 40.7% were mechanically ventilated and ICU mortality was 7.8%. ICU length-of-stay was 3.4(5.1) (median 1.8, range (0.17-60)) days and demonstrated marked kurtosis and right skew (29.4 and 4.4 respectively). BIC showed considerable spread, from a maximum of 509801 (OLS-raw scale) to a minimum of 210286 (LMM). R^2^ ranged from 0.22 (LMM) to 0.17 and the CCC from 0.334 (LMM) to 0.149, with MAE 2.2-2.4. Superior residual behaviour was established for the log-scale estimators. There was a general tendency for over-prediction (negative residuals) and for over-fitting, the exception being the GLM negative binomial estimator. The mean-variance function was best approximated by a quadratic function, consistent with log-scale estimation; the link function was estimated (EEE) as 0.152(0.019, 0.285), consistent with a fractional-root function.

**Conclusions:**

For ICU length of stay, log-scale estimation, in particular the LMM, appeared to be the most consistently performing estimator(s). Neither the GLM variants nor the skew-regression estimators dominated.

## Background

Length of stay during an intensive care unit (ICU) or hospital admission is a function of diverse patient and organisational input variables [1]. It is widely used as an indicator of performance [2] and is a determinant of costs, although resource allocation is also known to affect length of stay [3]. Not surprisingly, ICU length of stay has been the subject of frequent analysis [4-9], with the majority of studies presenting cross-sectional analyses over a relatively short periods of months [10] to 1–2 years [9].

ICU patient length of stay (and costs) demonstrate skewed distribution and various statistical modelling strategies have been employed in analysis of such data [11-14]; albeit linear regression (ordinary least squares regression, OLS) of the logged dependent variable has demonstrated a remarkable persistence [15]. Individual patient data, as accessed from ICU data-bases, have an intrinsic hierarchical structure (patients within ICUs) and due analytic consideration of this structure is also appropriate [16]. Using such data from the Australian and New Zealand Intensive Care (ANZICS) adult patient database (APD) [17], calendar years 2008–2009, the purpose of this paper was to: (i) compare the ***predictive performance and model specification*** of conventional estimators for skewed data (ICU length-of-stay); OLS (with both raw and log-scaled dependent variable) and generalised linear models (GLMs [14]), with more innovative approaches: multilevel or hierarchical linear mixed models (LMM) incorporating random effects [11,16]; extended generalised linear models (EEE) with flexible link and variance functions [18]; estimators utilising skew-normal and skew-*t* multivariate distributions [19]; and finite mixture (FMM) models which consider the dependent variable as a mixture of distributions [20-22]; and (ii) determine the ***effect of (ICU) mortality outcome upon length of stay***, allowing for “endogenous variable bias” [23] by means of a treatment-effects model [24,25] where specific allowance is made for the correlation of independent predictors and error terms. Under such conditions, model coefficients are biased [26].

## Methods

The ANZICS adult patient database (APD) is a bi-national (Australia and New Zealand) voluntary data collection of individual ICU admissions which commenced in 1990. The data set requirements are specified in a data dictionary [27]. The current data-base does not incorporate coronary care units. Data is collected at the individual ICU and uploaded to the central repository (ANZICS APD) for processing and quality assurance; which consists of a cycle of error and exception checks, site feed-back, resubmission and incorporation into a final reporting data-set.

The ANZICS APD was interrogated to define an appropriate patient set, over the time period 2008–2009. Physiological variables collected were the worst in the first 24 hours after ICU admission. All first ICU admissions to a particular hospital for the period 2008–2009 were selected. Exclusions: patients with an ICU length of stay ≤ 4 hours; and patients aged < 16 years of age. Specific attention was directed to the fidelity of severity of illness records; in particular the scoring of the Glasgow Coma Score (GCS). Records were used only when all three components of the GCS were provided. Records where all physiological variables were missing were excluded and for the remaining records, missing variables were replaced to the normal range and weighted accordingly [28]. ICU length of stay, initially recorded in hours was transformed to days; patients with an ICU length of stay > 60 days were not considered, no formal trimming methods were employed [29].

### Statistical methods

Continuous variables were reported using mean (SD), except where otherwise indicated; categorical variables were analysed using the Chi-squared test. To avoid the confounding effect of calendar time, patient data over the two years 2008 and 2009 was pooled. Distributional form of continuous variables of interest was by means of histogram, hanging rootogram [30,31] or violin plots [32]. The “hangroot”, an alternative to the histogram, compares an empirical distribution with a theoretical distribution by means of “hanging” spikes from the theoretical distribution. The spike lengths represent bin counts, using the square root of the frequencies to stabilise the sampling variation across bins and make deviations in the tails, where the counts are small, more visible. Deviations are shown as deviations from a horizontal line (*y* = 0) instead of deviations from a curve (the density function) in order to facilitate identification of patterns of deviation. The violin plot is a modification of the box plot that adds plots of the estimated kernel density to the summary statistics displayed by box plots [33], incorporates a marker for the median of the data, a box indicating the interquartile range, and spikes extending to the upper- and lower-adjacent values; overlaid is a density plot. Kernel density estimates are modifications of the histogram (a “smoothed” histogram), where densities are the continuous analogues of proportions (derivatives of the cumulative distribution function, so that areas under the density function read off as probabilities) [34]. Kurtosis (a measure of the heaviness of the tails of the distribution) and skewness were quantitatively expressed (a normal distribution having a skewness of 0 and a kurtosis of 3) according to standard formulae [35].

Predictor variables considered were

a) Continuous: age (in years), severity of illness score (Acute Physiology and Chronic Health Evaluation (APACHE) III score [36]); both centred for computation. The predictive effect of these variables was entered initially as both linear and quadratic.

b) Categorical: these were parameterized as indicator variables with the reference level (≡ 0) indicated in parentheses in the following list: gender (female); mechanical ventilation (not ventilated); ICU level, as defined in the ANZICS database, as Rural/Regional, Metropolitan, Tertiary and Private (Tertiary); State of origin; that is New Zealand and the States of the Commonwealth of Australia (New South Wales (NSW), the largest contributor); patient surgical status as post-elective surgery, post-emergency surgery and non-surgical (non-surgical); descriptors of ICU admission primary organ system dysfunction, these being a consolidation of the “diagnostic categories” of the APACHE algorithms: cardiovascular, gastrointestinal, metabolic, neurologic, respiratory, trauma, renal/genitourinary (cardiovascular); mean annual ICU volume across all ICUs (0 < mean volume; 1 > mean volume); Died-in-ICU (0 = Alive, 1 = Died)

Clinically meaningful combinations of variables and their interactions were assessed for effect; to preserve clinical transparency, higher order interactions were explored, but not entertained in the final model. The potential for multiple colinearity was tested using the variance inflation factor (VIF) and condition number (CN); where VIF < 10 and CN less than “30 or more” [37] were desirable.

ICU length of stay was modelled (with a fixed covariate list, and clustering on ICU site with robust variance [38]) via the estimators;

1. OLS using length of stay as a raw and a logged dependent variable. For the log OLS the expected value, *E*(*y*), is proportional to $\text{exp}\left(x^{'}\beta \right)$ and the variance is assumed to be proportional to the mean squared. Prediction is on the log-scale (the geometric mean) and re-transformation is dependent upon the distribution of the error term: if normally distributed $\hat{u}\left(x_{i}\right)=\text{exp}\left(x^{'}\hat{\beta }\right)+0.5{\hat{\sigma }}_{\varepsilon }^{2}$[39,40]; under homoscedasticity $\hat{\mu }\left(x_{i}\right)=\hat{\phi }.\exp\left(x_{i}^{'}\hat{\beta }\right)$ where $\hat{\phi }$ is the estimated smearing factor and is usually between 1 and 4 [41,42].

2. LMM (logged length of stay) using maximum likelihood for model estimates. Potential modifying covariates were computed as fixed effects; ICU-year units as random intercepts (or “levels”) and random coefficients (“slopes”: APACHE III and APACHE III squared, age and ventilation status) were incorporated into the model fit.

3. A treatment effects model, log-dependent variable, via the Stata™ module “treatreg” as initially described by Cong and Drukker [43]. A treatment-effects model is a two-equation system estimator (non-linear probit [44] and linear OLS), in which the effect of an endogenous binary variable (in this case, “Died-in-ICU”) on the continuous dependent variable is estimated by maximum likelihood [24,45]. Formally, the model is expressed in two equations [43]: the regression equation $y_{i}={\text{x}}_{i}\beta +\delta z_{j}+\varepsilon_{j}$, where z_*j*_ is the endogenous dummy variable indicating treatment assignment (=1, if $z_{j}^{*}>0$; or not, $z_{j}^{*}=0$). The outcome treatment-effect, estimated by the probit equation, is understood as being determined by an unobservable latent variable ($z_{j}^{*}$), a linear function of covariates (w_*j*_), and a random component (*u*_*j*_): $z_{j}^{*}={\text{w}}_{j}\gamma +u_{j}$. The covariates entered into the outcome treatment equation were: age mechanical ventilation status, APACHE III score and its square, patient surgical status and the interaction with ventilation status, descriptors of ICU admission primary organ system dysfunction. As these covariates were commensurate with those of the OLS equation, a question of model identification arises [25]. However, as shown by Maddala, such a model is identified even if the error terms are not independent and the variable list is identical in the two equations [45].

4. GLMs [14] with a log link (relating the conditional mean to the covariates) and either a gamma, inverse Gaussian or Poisson family (specifying the relationship between the variance and the mean) [14,42] using maximum likelihood. The possibility of over-dispersion with the Poisson GLM was tested by calculating $z=\frac{{\left(y_{i}-{\hat{\mu }}_{i}\right)}^{2}-y_{i}}{{\hat{\mu }}_{i}\sqrt{2}}$ and regressing *z* as a constant-only model; significance of *z* indicating over-dispersion [46]. In the presence of over-dispersion, the Poisson GLM model prediction and performance measures were replicated using a GLM negative binomial (NB2) model with log link [47]. For this model the variance function is given as $\mu +\alpha \mu^{2}$, where μ is the mean and α the heterogeneity or over-dispersion parameter, estimated by maximum likelihood (in GLM Poisson regression, α=0). The negative binomial may be also be conceptualised as a “waiting time” distribution (the number of days to discharge), a generalisation of the geometric distribution [5].

5. EEE model allowing simultaneous estimation of flexible parametric link (Box-Cox function [48]) and variance functions, as implemented by Basu in the Stata™ user-written module “pglm”, using the raw dependent variable, scaled to the mean [18,49]. Expected values, conditional on covariates, are an inverse Box-Cox function of the linear predictor: $x^{'}\beta =g\left(u_{i};\lambda \right)=\left(\mu_{i}^{\lambda }-1\right)/\lambda \text{ if }\lambda \neq 0\text{;}\;\text{log}\left(\mu \right)\;\text{if }\lambda =0$. The default power variance function was used, which sets the family of variance functions $\left(h\left(\mu_{i};\theta_{1};\theta_{2}\right)\right)$ to $\theta_{1}\mu_{i}{}^{\theta_{2}}$. The variance functions include the Poisson, gamma and inverse Gaussian variance functions as special cases [50]; as with linear OLS, where $\lambda =1\;\text{and }\theta_{2}=0$[51]. Estimation of regression and link parameters is via an extension of quasi-likelihood, and the variance parameters by additional estimating equations; that is, the EEE is a semi-parametric model. The link function transforms the mean of the outcome (not the outcome), overcoming the retransformation problem [50].

6. Regression utilising the skew-*t* and skew-normal distribution as implemented by Marchenko and Genton in the Stata™ user-written modules “skewtreg” and “skewnreg” respectively [19]. These distributions, fitted to the transformed (log) dependent variable, are (equivalently) the log-skew-*t* and log-skew-normal distributions by virtue of the correspondence which connects the normal and log-normal distributions [52,53]. The skew-normal distribution, beside location and scale parameters, has an additional shape parameter (α; if α = 0 ≡ normal distribution) regulating distributional asymmetry; the skew-*t* distribution allows for asymmetry and heavier tails via shape (α) and degrees-of-freedom (*v*) parameters [19]. Regression and other model parameters are estimated by maximum likelihood.

7. FMM, with two mixing components of a gamma and negative binomial (NB1 and NB2) densities, using the user-written Stata™ module “fmm”, version 2.0.0, by Deb [54]. In a FMM, the random variable is assumed to be drawn from a super-population that is an additive mixture of distinct subpopulations or classes (*C*) in proportions $\pi_{1},\ldots \pi_{C}\text{,}$where $\sum_{j=1}^{C}\pi_{j}=1\text{, 0<}\pi_{j}<1\left(j=1,\ldots C\right)$[20-22]. The *C*-point finite mixture model is given by $f(y_{i}|\Theta )=\sum_{j=1}^{c}\pi_{i}f_{j}(y_{i}|\theta_{j})$, where the mixing probabilities π_j_ are estimated with other parameters (Θ) [20-22]. The *θ*_*j*_ is a parameter vector characterizing the component density function f_j_(), the component density belonging to different parametric families (implemented in “fmm” are gamma, lognormal, negative binomial, normal, Poisson and Student-*t*). Estimation is by maximum likelihood.

Model adequacy was gauged by (i) progressive reduction in AIC (Akaike Information Criterion, for nested models) and BIC (Bayesian Information Criterion, for non-nested models) [55], both of which are penalised (with respect to number of observations and model parameters) likelihood methods for model determination (ii) likelihood ratio tests where appropriate and (iii) normality and lack of heteroscedasticity of residuals based upon graphical analysis [14]. Residuals were generated from the fitted model according to the scale of estimation [50]. The final model was developed on a determination set (80% of data) and validated on a validation set (20% of data); the random samples being stratified by (the 2) calendar-years. Predicted values were generated with continuous covariates centred and categorical covariates held at the reference category, as above, and in the presence of a logged dependent variable (length of stay) back-transformation to the original metric (days) utilised Duan’s smearing estimate [41]. Distributional form of the raw-scale predictions (determination and validation) were displayed using violin plots. Final model performance was assessed by R^2^ (on the “day” scale [56]) computed as the square of the correlation between predicted and observed length of stay [57]; Lin’s concordance correlation coefficient (between raw and predicted raw length of stay [58]); mean absolute error (MAE); and root mean squared error (RMSE) [12,14]. Supplementary diagnostic analyses [15,40,50,51,59] were also performed, using:

(i) user-written Stata™ code provided by Norton [60] and Jones [61]; the “GLM family” test (in GLMs there is accommodation of skewness, in particular, via the variance [42]: Var*y*|*x* = α.[E(*y*|*x*)]; therefore test if γ = 0 (Gaussian), = 1 (Poisson), = 2 (gamma), and = 3 (inverse Gaussian)); and the “COPAS” test: testing for over-fitting via split sample (50:50) cross-validation (1000x) using a version of the test initially formulated by Copas [62]; forecast predictions are regressed on raw-scale length of stay and a significant difference of β_predictions_ from unity suggests over-fitting [42]).

(ii) With respect to the particular problem that raw-scale variance was a power function of the raw-scale mean function, the (modified [40,59]) Park test [63] was implemented. Squared residuals from a provisional model (GLM or OLS log-scale) were regressed (using a GLM with robust variance estimate and log-link) on the predictions ($\hat{y}$) from the same model, both log transformed: $\text{ln}\left({\left(y_{i}-{\hat{y}}_{i}\right)}^{2}\right)=\gamma_{0}+\gamma_{1}\text{ln}\left({\hat{y}}_{i}\right)+v_{i}$; the coefficient on γ(see (i), above) indicating the GLM variance function.

(iii) a modified Hosmer-Lemeshow goodness-of-fit (*F*) test [50,64], regressing the particular model residual distribution against deciles of the linear predictor (using OLS with robust variance). Graphical plots of the calibration of regression decile-parameter estimates against deciles of the linear predictor were undertaken; positive parameter estimates above the null-line (≡ 0) indicate under-prediction and negative estimates indicate over-prediction [50].

As the interpretation of β coefficients is not invariant to model link functions [18], the average marginal effect [65,66] of ICU mortality upon length of stay (“Died-in-ICU”) was estimated using the “margins” routine of Stata™ and the specific computations provided by Basu [49] for the EEE estimator. A marginal (“partial” or “incremental” [18]) effect measures the effect on the conditional mean of the regression dependent variable (“*y*”, ≡ ICU length-of-stay) of a change in a regressor(s) of interest (in this case the binary variable “Died-in-ICU”) [66]. The impact of progressive increases in patient severity of illness (APACHE III score) was elucidated by the “marginsplot” module of Stata™ [67], using the contrast across the 1 (≡ Died)/0 (≡ Alive) binary variable “Died-in-ICU”. Because of the technical problem of applying the Duan estimator directly to a contrast of the predicted logs in the log-scale estimators (exponentiating the difference and then multiplying by a function of the residuals would not produce an estimate directly comparable to the contrast produced after regression performed on raw-scale data), the illustrative plots were generated using raw-scale OLS and a log-link Gaussian family GLM.

Stata™ (Version 12 MP, 2011; Stata Corporation, College Station, Texas) statistical software was used.

## Results

**Figure 1:**
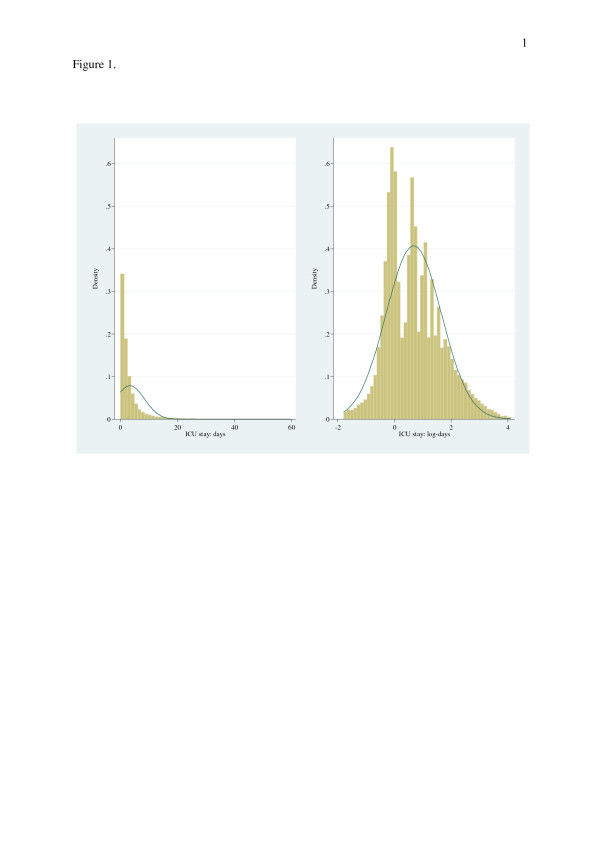
Histograms of ICU length of stay with normal curve overlay: left panel, raw scale; right panel, log transform.

**Figure 2:**
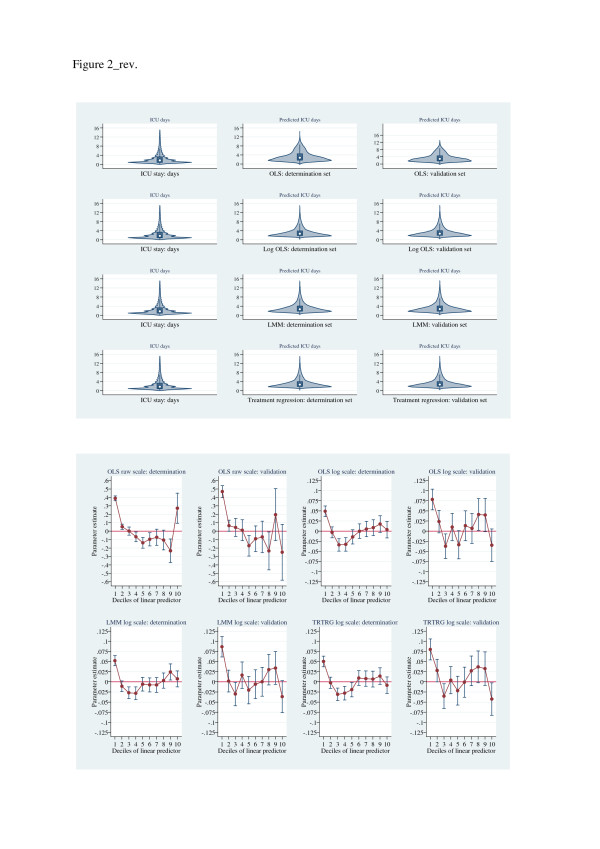
**Hanging rootograms displaying approximations of the empirical distribution of ICU length of stay, and transformations (log and square-root), to various theoretical distributions.** Shaded areas a tip of spiles are 95% CI.

The database for the period 2008–2009 contained records of 114798 patients with exclusion (4.20%) of patients having: ICU length of stay < 4 hours, incomplete GCS or APACHE III records. The final data set had missing ICU length of stay in 0.03% and 141 patients (0.001%) with ICU length of stay > 60 days (mean 81.98(24.37), range 60.05-199.67) not considered in analysis. Patients (*n* = 111663) admitted to 131 ICUs in the *same* number of hospitals, were of mean(SD) age 60.6(18.8) years, APACHE III score 52.5(28.9); 43.0% were female, 40.7% were mechanically ventilated (on the day of admission) and ICU mortality was 7.8%. ICU length of stay was 3.4(5.1) (median 1.8, inter-quartile range 2.8 (0.93-3.7)) days. Summary statistics of length of stay, APACHE III score, age and gender by ventilation status and patient surgical type are shown in Table 1. Overall ICU length of stay and the log transform are displayed in histogram form with normal curve overlay in Figure 1 (left and right panels respectively); raw scale length of stay demonstrated marked kurtosis and right skew (29.4 and 4.4 respectively) whilst the log transform demonstrated a more symmetrical distribution (kurtosis 3.1 and skewness 0.5). Comparison of the empirical distribution of ICU length of stay and its’ log and square root transformation showed poor approximation to standard theoretical distributions (normal, lognormal and gamma; Figure 2).

**Table 1:** Demographics of ICU-hospital level, ventilation and surgical status: mean(SD)

**ICU hospita**l	**Overall**		**Not-ventilated**			**Ventilated**	
**Level/variable**		**Non-surgical**	**Elective surgical**	**Emergency surgical**	**Non-surgical**	**Elective surgical**	**Emergency surgical**
***Rural***	***(13.05%)***						
ICU length of stay (days)	3.3 (4.6)	2.6 (3.1)	2.2 (2.6)	2.5 (3.6)	5.7(7.1)	4.7 (4.5)	5.4 (6.4)
APACHE III score	49.3(29.2)	42.6 (23.9)	36.4(16.1)	40.8 (20.8)	78.4 (33.6)	54.7 (25.3)	70.5 (30.8)
Age (years)	60.1 (19.5)	58.5 (20.4)	63.8 (16.1)	61.3 (20.5)	57.9 (18.3)	67.8 (15.1)	64.0(19.0)
% males	55	55	56	51	58	66	56
***Metropolitan***	***(18.45%)***						
ICU length of stay (days)	3.7 (5.2)	3.1 (4.3)	1.8 (2.2)	2.3 (3.4)	5.7 (6.8)	4.7 (5.8)	5.3 (6.6)
APACHE III score	55.5 (30.6)	50.4 (26.3)	37.3 (16.7)	44.9 (23.6)	76.4 (34.5)	55.5 (23.1)	65.5 (29.7)
Age (years)	59.9 (19.2)	58.6 (19.9)	63.8 (16.1)	61.3 (20.5)	57.7 (19.2)	66.0 (15.2)	63.3 (19.2)
% males	55	52	58	50	56	66	59
***Tertiary***	***(44.49%)***						
ICU length of stay (days)	3.9 (5.7)	3.0 (4.3)	1.5 (1.8)	2.4 (3.9)	5.9 (7.2)	2.7 (4.1)	5.5 (6.7)
APACHE III score	57.0(30.1)	51.6 (26.1)	38.7 (15.8)	45.2 (22.7)	73.4 (34.6)	48.3 (19.0)	60.8 (27.8)
Age (years)	58.3 (19.2)	57.3 (20.1)	62.8 (16.2)	59.7 (20.1)	54.6 (19.6)	64.2 (15.6)	57.9 (19.9)
% males	60	55	60	56	62	63	62
***Private***	***(24.01%)***						
ICU length of stay (days)	2.4 (3.7)	3.2 (4.5)	1.5 (1.8)	1.9 (2.7)	6.6 (7.9)	3.2 (3.9)	4.5 (6.6)
APACHE III score	43.7(22.4)	49.6 (22.9)	35.3 (15.2)	40.5 (19.1)	78.1 (32.1)	52.3 (21.7)	62.48 (29.4)
Age (years)	65.5 (16.0)	68.4 (16.9)	63.4 (16.0)	64.6 (17.9)	68.9 (15.7)	68.4 (13.8)	67.9 (16.1)
% males	57	52	54	47	54	61	49

Comparative performance of the estimators for ICU length of stay is seen in Tables 2 and 3; the regression models had 52 fixed parameters, including the constant term. Over the range of measures assessing predictive performance and model specification (including goodness-of-fit), the LMM demonstrated a consistently better performance compared with the other 11 estimators; albeit all estimators revealed some problematic aspects of residual analyses. BIC for all the log-scale estimators (LMM, treatment effects- and log-skew-regressions) were considerably less than for estimation on the raw scale (Table 2). The final model variable set displayed a condition number of 19.4 and a mean VIF of 3.76; the best R^2^ and concordance correlation coefficient was 0.22 and 0.334 respectively.

**Table 2:** Performance comparisons for various estimators

**Performance index**	**BIC**	**R^2**	**R^2**	**CCC**	**CCC**	**MAE**	**MAE**	**RMSE**	**RMSE**	**Residual**	**Normality**	**Residual vs**
**Model**	**Determination set**	**Determination data-set (n = 86740)**	**Validation data-set (n = 21678)**	**Validation data-set**	**Determination data-set**	**Determination**	**Validation**	**Determination**	**Validation**	**P-P plot**	**Q-Q plot**	**fitted**
**OLS: raw scale**	509801.4	0.19	0.18	0.313	0.308	2.4	2.4	4.6	4.5	No	+ ve skew	No
**OLS: log scale**	216183.2	0.18	0.18	0.293	0.290	2.4	2.4	4.6	4.5	Yes	+ ve skew	Yes
**LMM (log scale)**	210285.8	0.22	0.20	0.334	0.328	2.3	2.3	4.5	4.5	Yes	+ ve skew	Yes
**(Raw scale)**	507492.8											
**Treatment effects regression (log scale)**	244883.2	0.18	0.17	0.296	0.294	2.4	2.4	4.6	4.5	Yes	+ ve skew	Yes
**(Raw scale)**	539291.4											
**GLM: family(Poisson), link(log)**	479143.3	0.19	0.18	0.321	0.314	2.4	2.4	4.5	4.5	No	+ ve skew	No
**GLM: family(negbin), link(log)**	375021.6	0.19	0.18	0.322	0.316	2.4	2.4	4.5	4.5	No	+ ve skew	No
**GLM: family(gamma), link(log)**	355449.9	0.19	0.18	0.322	0.317	2.4	2.4	4.6	4.5	No	+ ve skew#	No
**GLM:**	365186.1	0.19	0.18	0.322	0.317	2.4	2.4	4.6	4.6	No	+ ve skew#	No
**family(inverse Gaussian), link(log)**												
**EEE:**	Not estimable	0.19	0.18	0.320	0.316	2.4	2.4	4.6	4.6	No	+ ve skew#	No
**Log-skew-*t *regression**	213465.5	0.18	0.17	0.285	0.283	2.4	2.4	4.6	4.6	Yes	neg skew#	No
**(Raw scale)**	349461.3											
**Log-skew-normal regression**	214216.4	0.18	0.17	0.317	0.273	2.6	2.4	4.6	4.6	No	neg skew#	No
**(Raw scale)**	436227.8											
**FMM: raw scale**	329774.9	0.18	0.18	0.151	0.149	2.2	2.2	5.0	5.0	No	+ ve skew#	Yes

LLM; Linear mixed model (random coefficient model). CCC; concordance correlation coefficient. Negbin; negative binomial. P-P plot; standardized normal probability plot. Q-Q plot; plot of the quantiles of the residuals against the quantiles of the normal distribution . +ve skew#; marked positive skew. neg skew#; marked negative skew. Residuals vs fitted; increase spread of residuals with increment of fitted values (predicted length of stay). MAE; mean absolute error. RMSE; root mean square error. FMM; finite mixture model. R^2^, CCC,MAE and RMSE were computed on the back-transformed “day” scale (for the linear mixed model, OLS-log, “treatreg” log-skew-t and log-skew-normal regression) using the Duan smearing estimate.

**Table 3:** Specification tests for various estimators

	**Correlation**	**Residuals**		**Hosmer-Lemeshow (*F*) test**		**Copas test**
	***LP vs residuals***	***Skewness***	***Kurtosis***	**Determination**	**Validation**	
**OLS raw scale**^**@**^	1.00	4.33	32.74	0.0001	0.0001	0.996(0.993, 0.998)
**OLS log scale**^**@**^	0.03	0.25	3.58	0.0001	0.0001	1.079(1.063, 1.094)
**LMM log scale**^**@**^	0.008	0.25	3.76	0.0001	0.0001	1.054(1.036, 1.072)
**Treatment effects model: log scale**	0.150	0.27	3.58	0.0001	0.0001	1.043(1.025, 1.062)
**GLM: family(Poisson), link(log)**^**#**^	0.0001	2.37	13.41	0.0001	0.0001	0.940(0.899, 0.981)
**GLM: family(negative binomial), link(log)**^**#**^	0.0001	1.59	8.14	0.0001	0.0001	0.967(0.926, 1.008)
**GLM: family(gamma), link(log)**^**#**^	0.0001	1.26	6.60	0.0001	0.0001	0.923(0.882, 0.965)
**GLM: family(inverse Gaussian), link(log)**^**#**^	0.0002	0.20	4.98	0.0001	0.0001	0.776(0.717, 0.835)
**EEE: raw scale**	0.002	4.25	32.44	0.0001	0.002	0.956(0.957,0.958)
**Skew-*t *regression: log scale**	0.0001	0.31	3.59	0.0001	0.0001	1.085(1.065, 1.105)
**Skew normal regression: log scale**	0.0001	0.32	3.56	0.0001	0.0001	1.134 (1.113, 1.155
**FMM: raw scale**	0.0001	4.68	33.70	0.0001	0.0001	0.795(0.737, 0.852)

LP, linear predictor. ^@^, standardised residuals [13]. ^#^, Deviance residuals [68].

**Figure 3:**
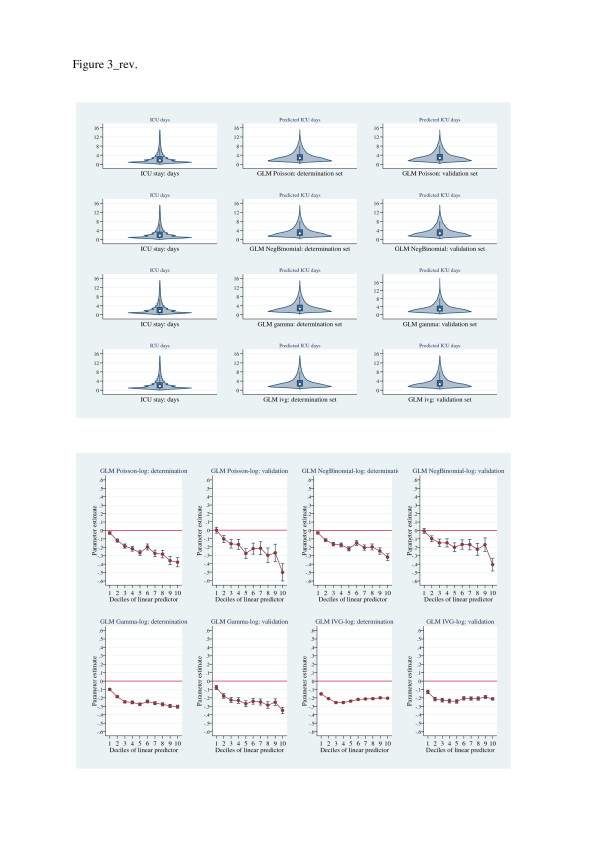
Histograms of predicted mean ICU days for component 1 (left panel) and component 2 (right panel) of the FMM.

The outcome discrimination (Died-in-ICU) of the probit sub-equation of the treatment effects regression was excellent for both determination and validation sets (ROC curve area 0.92). The null hypothesis, that the correlation (*ρ*=0.065) between the error terms of the separate estimations is zero, was rejected at P = 0.0001. The Poisson GLM exhibited overdispersion (*P* value for “z” = 0.0001), the negative binomial GLM demonstrating an expected decrease in BIC; other performance measures were comparable between these two models. However, over-fitting, as assessed by the Copas test, was demonstrated in all other models except for the negative binomial GLM (Table 3). The “GLM family test” of the variance function of GLMs (Var*y*|*x* = α.[E(*y*|*x*)]γ [60]) rejected tests of γ for 0, 1, 2 and 3 (*P* ≤ 0.0001). The modified Park test generated estimates for γ of: 0.348(0.333, 0.364) for log-scale OLS; 1.659(1.609, 1.708) for GLM Poisson-log; 2.106(2.005, 2.207) for GLM gamma-log; and 2.352(2.088, 2.616) for GLM inverse Gaussian-log. The EEE link parameter (λ) estimate was 0.152(0.019, 0.285) and the variance function (θ_2_) was 2.115(2.013, 2.218). The log-skew-normal and log-skew-*t* regressions reported α values of 1.073(0.933, 1.213) and 1.490(1.323, 1.659) respectively, suggesting positive-skew; the degrees-of-freedom for the log-skew-*t* regression was 10.53(7.97, 13.91) implying heavier-than-normal tails for the conditional distribution of (log) ICU length-of-stay. The 2 component FMM gamma model generated predicted mean ICU days of 0.56(0.32), range 0.06, 3.64 (component 1) and 3.69(2.45), range 0.49, 19.1 (component 2) as illustrated in Figure 3. The FMM negative binomial models demonstrated non-convergence.

**Figure 4:**
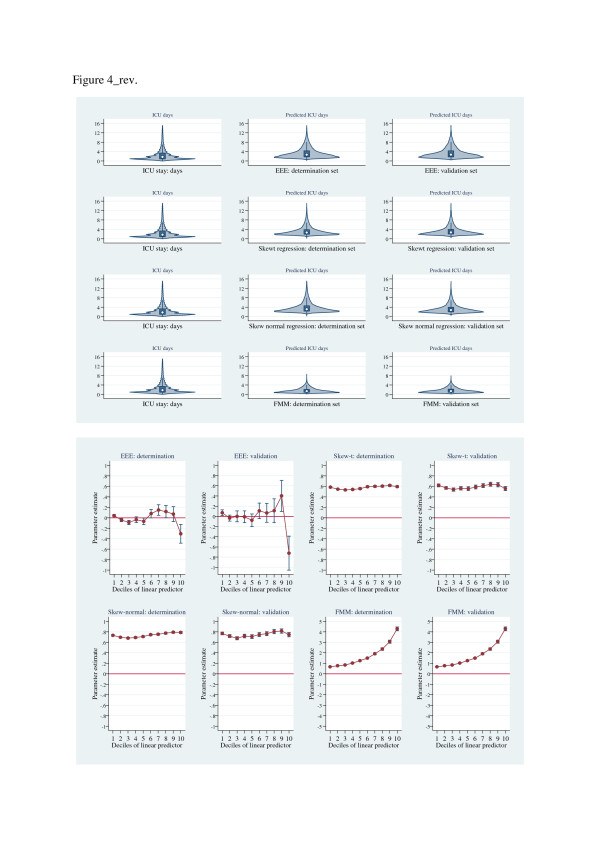
**Upper panel: Violin plots of ICU length of stay (raw-scale, length of stay < 16 days) and predicted length of stay (raw-scale, < 16 days; determination and validation data sets) for estimators: OLS (raw-scale), log-scale OLS, LMM, and treatment effects regression.** Lower panel: Hosmer-Lemeshow (*F*) test of residuals across deciles of linear-predictor, for estimators: OLS (raw-scale), log-scale OLS, LMM, and treatment effects regression. X-axis, linear predictor; Y-axis, parameter estimates.

**Figure 5:**
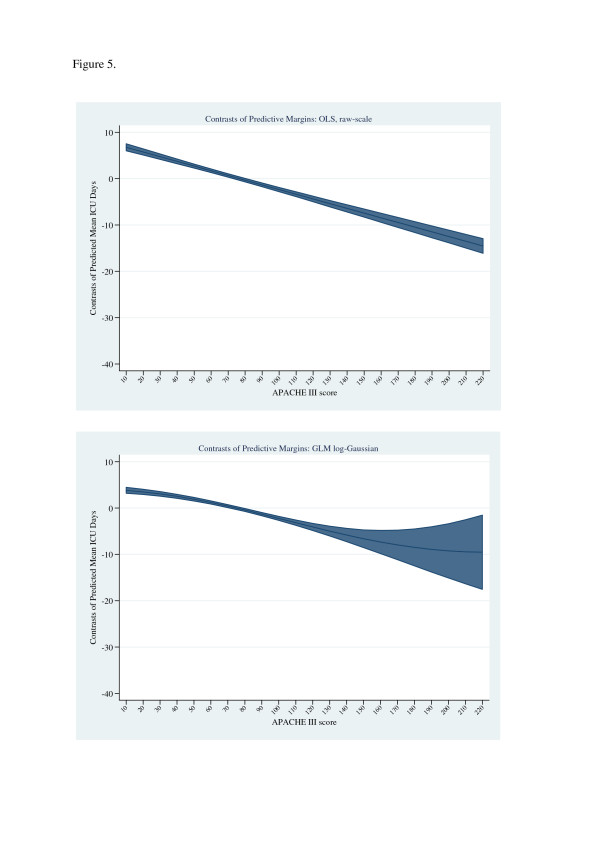
**Upper panel: Violin plots of ICU length of stay (raw-scale, length of stay < 16 days) and predicted length of stay (raw-scale, < 16 days; determination and validation data sets) for estimators: GLM Poisson-log, GLM negative binomial-log, GLM gamma-log, GLM inverse Gaussian-log.** Lower panel: Hosmer-Lemeshow (*F*) test of residuals across deciles of linear-predictor, for estimators: GLM Poisson-log, GLM negative binomial-log, GLM gamma-log, GLM inverse Gaussian-log. X-axis, linear predictor; Y-axis, parameter estimates. determin., determination. Validat., validation.

**Figure 6:**
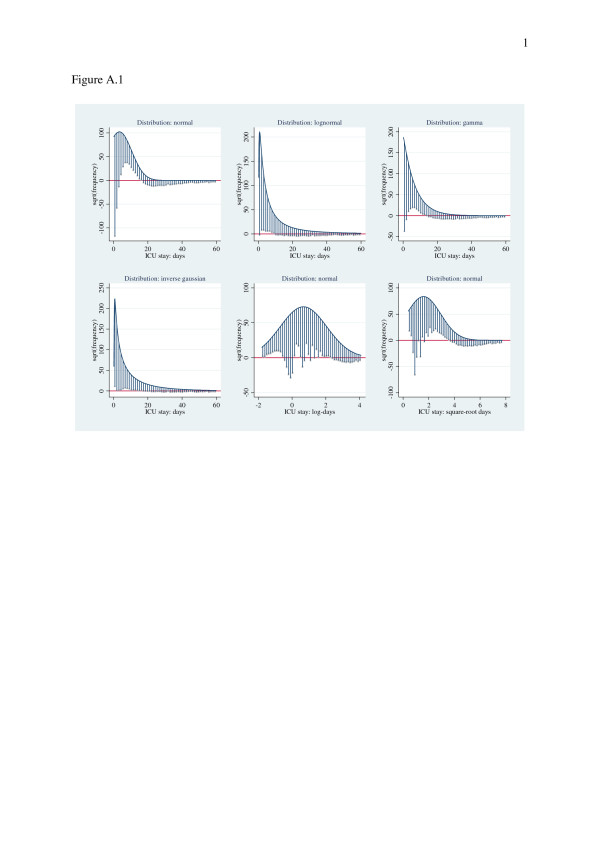
**Upper panel: Violin plots of ICU length of stay (raw-scale, length of stay < 16 days) and predicted length of stay (raw- scale, < 16 days; determination and validation data sets) for estimators: EEE, log-skew-*t *regression, log-skew-normal regression and FMM.** Lower panel: Hosmer-Lemeshow (*F*) test of residuals across deciles of linear-predictor, for estimators: EEE, log-skew-*t* regression, log-skew-normal regression and FMM. X-axis, linear predictor; Y-axis, parameter estimates.

Figures 4, 5, 6 show (i) upper panels: violin-plot distributional form of predictions and (ii) lower panels: Hosmer-Lemeshow-test parameter estimates, for determination and validation sets for each of the estimators, grouped by increasing scalar values of the Hosmer-Lemeshow parameter estimates. The graphical displays show a progressive lack-of-fit (Hosmer-Lemeshow test estimates) across Figures 4, 5, 6 in concert with corresponding over- and under-prediction as revealed by the violin plots. The LMM, log-scale OLS and treatment effects regression were the best calibrated; the EEE showed good calibration across the linear predictor deciles except for the upper deciles where substantial discordance was evident.

**Figure 7:**
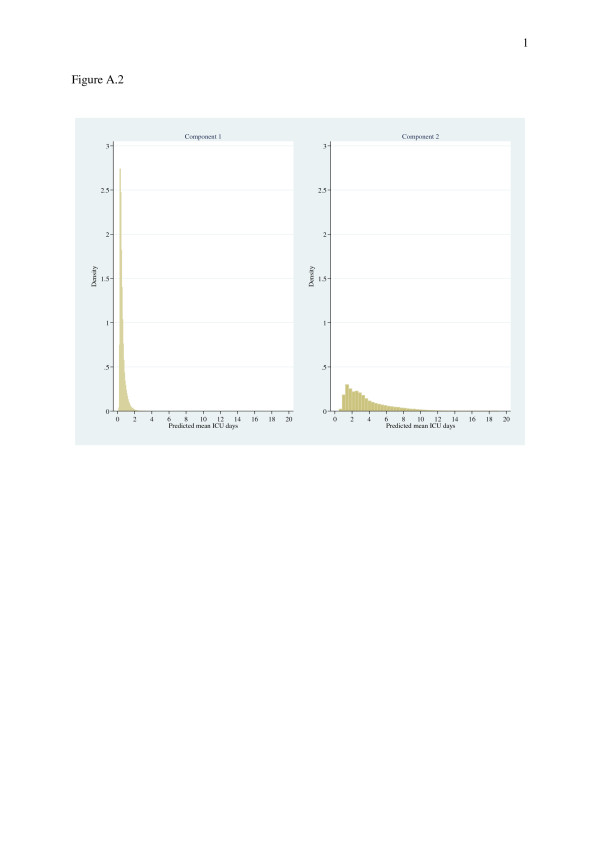
Contrasts of predictive margins (ICU days) across the binary variable “died in ICU” for OLS raw scale (upper panel) and GLM, Gaussian family and log link (lower panel).

The β regression estimates for the binary variable “Died-in-ICU” are seen in Table 4, final column, and range from a low of 29% (treatment effects regression) to 295% (raw-scale OLS).The average marginal effects are also tabulated, and were generally decreased in magnitude compared with the β estimates. Figure 7 illustrates the contrasts of predictive margins for “Died-in-ICU” across increments of APACHE III score for raw-scale OLS and log-link Gaussian family GLM (see also Table 4). Similar decreases (not shown) of the contrasts of predictive margins across the APACHE III score range were evident for the (log) linear predictor for estimators using log-ICU length of stay.

**Table 4:** β regression estimate and average marginal effect for “Died in ICU”

	**β estimate**	**Average marginal effect**
OLS raw scale	2.947 (2.368, 3.526)	2.486 (2.060, 2.912)
OLS log scale	0.404 (0.302, 0.507)	0.353 (0.268, 0.437)
LMM log scale	0.367 (0.318, 0.416)	0.320 (0.285, 0.355)
Treatment effects regression: log scale	0.297 (0.194, 0.401)	0.215 (0.125, 0.302)
GLM: family(Poisson), link(log)	0.600 (0.444, 0.676)	1.456 (1.064, 1.848)
GLM: family(negative binomial), link(log)	0.596 (0.476, 0.715)	1.679 (1.239, 2.119)
GLM: family(gamma), link(log)	0.619 (0.497, 0.740)	1.802 (1.336, 2.269)
GLM: family(inverse Gaussian), link(log)	0.757 (0.608, 0.906)	2.648 (1.914, 3.382)
EEE: raw scale	0.678 (0.539, 0.817)	1.507 (1.042, 1.972
Skew-*t* regression: log scale	0.376 (0.268, 0.483)	0.335 (0.250, 0.420)
Skew normal regression: log scale	0.314 (0.214, 0.413)	0.271 (0.195, 0.348)
FMM: raw scale, component 1	0.234 (0.121, 0.347)	0.130 (0.066, 0.195)
FMM: raw scale, component 2	0.648 (0.513, 0.784)	2.395 (1.871, 2.920)
GLM: family(Gaussian), link(log)	0.541 (0.421, 0.662)	1.285 (0.902, 1.667)

## Discussion

Methodological recommendations for non-normal data analysis have usually been subsumed under the rubric of cost analysis [12,15,69]. The distribution of positive health expenditures (and length of stay) exhibit skew, kurtosis and heteroscedasticity, with a non-constant and increasing variance, albeit particular cost problems, such as the accommodation of a probability mass at “zero”, may not have immediate relevance to length of stay analysis [51,69]. This review has suggested that a LMM had better overall performance compared with 11 other estimators. However, a number of issues pertain to comparator studies of possible estimators; in particular data structure and the full assessment, or otherwise, of model specification.

The hierarchical nature of some clinical and/or administrative data sets and the requirement for appropriate analysis has been commented upon above. As opposed to the volume-outcome literature [70], mixed models would appear to have been have been applied in a relatively small number of studies for length of stay analysis [16,71-75]. The use of administrative data [5,16] may also be associated with integer based calendar–day recording which lacks the accuracy of fractional day based “exact” times [10,71].

Because of the non-normality of length of stay, formal trimming [76,77] or truncation [71,77,78] of the data, or deleting [5,73,79] “outliers” has been undertaken prior to possible data transformation. For instance, studies implementing the APACHE III [78] or IV [71] algorithms for predicting ICU length of stay have truncated the latter at 30 days, at a 1% data level; the same fraction as seen with data deletions [73]. The current study used a deletion fraction of 0.001%. The motivation for such strategies are various [80], but the theoretical basis of these data revisions has been questioned [29] and bias in the estimation of the mean has also been suggested [29,69]. More importantly, large values of length of stay are “true” values [69] and data reduction cannot necessarily be defended as “…represent[ing] unusual cases…”, or because of “…analytical problems…” [73]. That trimming will cause decreases in residual variance in LMM was pointed out by Lee et al. [72] and was easily demonstrated in the current data set; where the residual variance was 0.646 for the full data, 0.600 at the 99^th^ percentile trim (< 26 days) and 0.508 for the 95^th^ percentile trim (< 12 days) of ICU length of stay. Model assessment must take note of data revisions.

Similarly, the repeated assertions [71,72,76,77,80,81] that the objective of such data manipulations are model (in particular, OLS) requirements for normality of the dependent variable are problematic. As observed by Buntin and Zaslavsky “Plotting of the data with various transformations is thus a useful preliminary step, but ultimately the distribution of the residuals from the transformed model is critical because the model assumptions concern the distribution of the residuals, not of the data” [59]. That the residuals “…usually have a similar distribution to the original data…” [12] does not absolve the analyst from model based residual interrogation; the simple reporting of overall measures such as R^2^[77] or agreement indices of observed versus predicted [71] are not satisfactory proxies. Moreover, inference on β-coefficients is biased under heteroscedasticity.

Effect of ICU death on length of stay.

The β coefficient for “Died-in-ICU”, as a main effect, ranged from +29.7% (treatment effects regression, log scale) to +300% (OLS, raw scale), consistent with the β estimates for the factor “Death” of +0.536 (age < 65 years) and +0.439 (age ≥ 65 years) reported by Angus et al. [5], using a GLM (geometric family, identity link). However, the results of the marginal analysis (Table 4) implied over-prediction of the β-coefficient of “Died in ICU” across the estimators, albeit the average marginal effect was considered, as opposed to the marginal effect at the mean or at a particular value [65,66]. Furthermore, there was a progressive decrease in the contrast, died in ICU versus alive in ICU, of predicted mean ICU days with an increase in the APACHE III score (Figure 7); this effect being consistent with findings from our previous study [82] and those of Rapaport et al. [8] and Woods et al. [9]. Of more interest was the demonstration, using the treatment effects regression model, that the covariate “Died-in-ICU” (which also has the status of an “outcome”) was associated with a significant correlation (*ρ*) between the error terms of the separate estimations (probit and linear regression) and was appropriately considered as endogenous [26]. That *ρ* was positive at 0.065 also indicated that OLS overestimated the “treatment” effect (Died-in-ICU); that is, a face-value interpretation of the β mortality coefficient was problematic. Endogeneity may also be suspected in mortality models where length of stay [7] or mortality probability [83] are entered as predictive covariates. Such regression of a variable upon its components has been termed a “dubious practice” [15]. This being said, the extension of 2-stage methods to non-linear models may not be without its own issues [15,84,85].

Estimators of length of stay.

The statistical basis of the various estimators used in the current paper has been canvassed in “Statistical methods”, above. These estimators, assessing the mean function $u\left(x\right)$, have seen extensive application and assessment in the cost data literature, but not necessarily for length of stay. The recent introduction of log-skew-normal and log-skew-*t* estimators has not afforded opportunity for extensive assessment. Within the ICU literature, the predominant estimators have been ordinary least squares regression, with [8] and without [7,71,78] log transformation; generalized linear model (geometric family with identity link) [5] and random intercept linear model [83]. Lee and co-workers have explored alternative approaches suited to the analysis of hospital length of stay [74,86].

It is perhaps not surprising that in a large hierarchically structured data set that the LMM appeared to dominate. The suggestion of lack of discriminatory power across alternative estimators in cost analysis, albeit with small sample size [15], also appears to be vindicated in the current analysis. However, the further suggestion that large sample size together with “…simple methods…” [69] and an identity link [12,15] may be analytically sufficient seems not to be the case with our data, although the LMM and log-OLS models were relatively easy to fit and interpret. The estimated link function (*λ*=0.152(0.019, 0.285) supports neither an identity (*λ*=1) nor log link (*λ*=0), rather a fractional- but not a square-root (*λ*=0.5) function. Although the Park test and the EEE estimation of the variance function approximated 2, the GLM variants demonstrated poor goodness-of-fit (Figure 5) with the exception of the EEE (not with-standing the upper most decile of the linear predictor, Figure 6) where the link and variance functions were estimated, not fixed. The narrow confidence limits in the Copas test (Table 3) for the EEE estimator presumably reflects this re-estimation of link and variance functions in the cross-validation process. The residual behaviour of (i) the log scale estimators (Table 3) was generally satisfactory and empirical estimation suggested a quadratic mean-variance relationship which was consistent with the log-OLS estimator and (ii) the GLM models, was variable and with heavy tails. The use of gamma distribution in the presence of heavy tails has recently been cautioned [69]. Estimators on the raw scale (OLS and FMM) demonstrated consistent but sub-optimal residual behaviour; and despite modest BIC values, the log-skew estimators had an overall poor residual performance.

In the current study, the range of R^2^ was modest (0.18-0.22; Table 2); consistent with that previously reported, 0.13 [78] to 0.21 [71,83], and the observations of Diehr et al. that R^2^ for cost utilisation data are usually ≤ 0.20 [12]. Increases in R^2^ have been reported, not surprisingly, across groupings; for instance, ICU units (R^2^ = 0.78; [7]). In the current study, R^2^ across ICU units (n = 118) was 0.92 (LMM, determination set).

### Critique of methodology

The hierarchical structure of data under analysis is not uncommon in clinical or administrative data sets, but may not be seen in cost utilisation studies where survey and administrative data predominate. Thus cross paradigm performance assessments may lack validity, although the skew and kurtosis indices (4.4 and 29.4 respectively, in the current data) at least were comparable (4.1 and 25.6 [42], 4.4 and 50 [50]). The significance of the residual tests (Table 3) and the tendency to over-fitting must be interpreted in the context of the large data sets [87] and the reported adverse effect of outliers and skewed data on these tests [15,42]. We chose not to benchmark goodness of fit against the behaviour of raw residuals from the log-scale estimators. This preference was dictated by the application of homoscedastic retransformation from log-scale estimation which may not have been optimal, leading to the generation of raw-scale residuals ($y_{i}-{\hat{y}}_{i}$) of uncertain status. Such uncertainty may be contrasted with the fixed relationship (via the inverse logit function) between the scale of estimation (log-odds) and the scale of interest (probability) in logistic regression where the Hosmer-Lemeshow test was first described. No generally applicable method of heteroscedastic retransformation recommends itself with large data sets and multiple predictor variables [39,42,51,59] and was not undertaken. Our analysis suggests that percentage changes in days are important, not absolute changes; this follows from the fact that the data satisfy important optimal statistical properties on the log scale, and that we can therefore estimate and interpret effects on this scale with some confidence.

Comparisons between estimators with respect to the significance (or otherwise), the effect magnitude and form (for continuous variables) of other covariates entered into the regressions (the covariates were fixed [11,59]) were not a focus of interest and were not reported. The relatively poor performance of the GLM model variants may be subject to enhancement by the use of generalised linear mixed models [88] which could incorporate the random effects structure of the LMM. Similarly, our use of the Poisson and negative binomial GLM fails to account for the structural exclusion of zero counts (both distributions include zeros) and use of zero-truncated count models may have been appropriate [47].

## Conclusion

Model choice under the conditions of the current data set would appear to favour the LMM and log-OLS. The treatment effects model afforded extra explanation with respect to the covariate “Died-in-ICU” and had a good fit to the data. Mechanistic approaches to estimation are represented by the FMM which captures the bi-modal nature of the data (Figures 1 and 3), although the fit was not optimal; and the negative binomial GLM which represented the length of stay as “waiting times”. Neither the log-skew-normal nor log-skew-*t* estimator would appear to recommend themselves as the preferred analytic approach for the current data set.

## Competing interests

The authors declare that they have no competing interests.

## Authors’ contributions

The study was conceived, designed, (data)-analysed, written and critically revised jointly by both authors (JLM, PJS). Both authors read and approved the final manuscript.

## Pre-publication history

The pre-publication history for this paper can be accessed here:

http://www.biomedcentral.com/1471-2288/12/68/prepub
